# Bilateral vas deferens suturing to prevent inguinal hernias after radical prostatectomy

**DOI:** 10.1002/bco2.70064

**Published:** 2025-08-06

**Authors:** Kenichi Hata, Yuma Goto, Masaki Hashimoto, Yusuke Takahashi, Yuki Takiguchi, Yuya Iwamoto, Shun Saito, Ayaka Kawaharada, Yuki Enei, Keigo Sakanaka, Kazuhiro Takahashi, Akira Hisakane, Taisuke Yamazaki, Keiji Yasue, Soichiro Aoki, Kanako Kasai, Takafumi Yanagisawa, Shunsuke Tsuzuki, Gen Ishii, Toshihiro Yamamoto, Hiroshi Sasaki, Jun Miki, Tatsuya Shimomura, Hiroki Yamada, Akira Furuta, Kenta Miki, Takahiro Kimura

**Affiliations:** ^1^ Department of Urology Atsugi City Hospital Atsugi City Kanagawa Japan; ^2^ Department of Urology Jikei University School of Medicine Tokyo Japan

**Keywords:** inguinal hernia, prophylaxis, prostate cancer, prostatectomy, vas deferens

## Abstract

**Objectives:**

To evaluate the efficacy and safety of bilateral vas deferens sutures in preventing postoperative inguinal hernia after prostatectomy for clinically localized prostate cancer.

**Materials and Methods:**

This retrospective study included 282 patients with localized prostate cancer who underwent open or laparoscopic radical prostatectomy between July 2012 and July 2023. The inguinal hernia incidence rates were compared between the vas deferens suture group (141 patients, May 2017 to July 2023) and the control group (141 patients, July 2012 to April 2017). We further determined the risk factors for inguinal hernia after prostatectomy using a multivariate regression analysis.

**Results:**

Among the 282 patients analysed, postoperative inguinal hernia was observed in 10 (7.1%) and 37 (26.2%) patients in the vas deferens suture and control groups, respectively. The incidence differed significantly between patients who did and did not undergo radical prostatectomy with a prophylactic procedure (*P* = 0.006). The 2‐year inguinal hernia‐free rates were 93.4% and 85.1% in the vas deferens suture and control groups, respectively. The median duration for inguinal hernia development was 15 months. Multivariate analysis identified the vas deferens suture procedure as a single factor associated with protection against inguinal hernia development after radical prostatectomy (hazard ratio, 0.36; 95% confidence interval, 0.177–0.734; *P* = 0.005).

**Conclusions:**

The vas deferens suture is a simple and safe prophylactic procedure to decrease the risk of inguinal hernia after radical prostatectomy.

**Abbreviations and Acronyms**
BMI: body mass index
IH: inguinal hernia
LRP: laparoscopic radical prostatectomy
PLND: pelvic lymph node dissection
P‐vol: prostate volume
RALP: robot‐assisted laparoscopic radical prostatectomy
RP: radical prostatectomy
RRP: retropubic radical prostatectomy

## 1 INTRODUCTION

Inguinal hernia (IH) repair is a common procedure, with more than 800 000 repairs performed annually in the United States. Approximately 25% of men develop IH during their lifetime.^1^ However, the reasons underlying IH development remain unclear, and epidemiologic data regarding the details of IH occurrence are limited.^2^ Increased intra‐abdominal pressure, such as that caused by obesity, chronic cough, constipation and benign prostate hyperplasia, is considered a risk factor for IH.^1^, ^3^ Various other risk factors, including family history, previous contralateral hernia, male sex, age, abnormal collagen metabolism, low body mass index (BMI), patent processus vaginalis and radical prostatectomy (RP), are also thought to be associated with IH occurrence in a complex manner.^4^

IH is a recognized complication of retropubic radical prostatectomy (RRP) for clinically localized prostate cancer.^5^ The reported incidence of IH post‐RRP ranges from 8.4% to 38.7%.^5^, ^6^, ^7^ Nilsson et al. assessed the incidence of IH repair after RP for prostate cancer, as compared with a control population, using the Prostate Cancer Database of Sweden. They analysed data from 28 608 patients who underwent RRP, minimally invasive RP (laparoscopic radical prostatectomy [LRP], robot‐assisted laparoscopic radical prostatectomy [RALP]) and radiation therapy for prostate cancer and compared them with 105 422 control individuals. The incidence of IH repair after RP was four‐fold higher in the RP group than in the control group. The incidence was slightly higher after RRP than that after minimally invasive RP. Moreover, there was a significant difference in the proportion of indirect IH at repair between men who had undergone RP and controls: indirect IH occurred in 77% of cases after RRP and 66% after minimally invasive RP. The odds ratio for an indirect IH in men who had undergone RRP compared with controls was 3.05.^8^

Various factors have been associated with IH development after RP. These include age, BMI, stream and straining prior to RP detected on the international prostate symptom score, subclinical IH, previous IH repair, patent processus vaginalis, surgeon experience, postoperative continence, Retzius space‐sparing surgery, nerve sparing and anastomotic stricture.^9^, ^10^ However, the mechanism of prostatectomy‐associated IH development remains unclear.

We hypothesized that the processus vaginalis migrates into the internal inguinal ring due to amputation of the vas deferens during RP, leading to postoperative IH. Thus, we considered that suturing the bilateral vas deferens, which had been fixed by the prostate, might prevent the processus vaginalis from being pulled into the inguinal canal, thereby preventing postoperative IH. To evaluate this hypothesis, we compared the incidence of postoperative IH in men who underwent RRP and LRP with and without vas deferens suturing (VDS) and sought to identify the risk factors for postoperative IH development.

## 2 METHODS

### 2.1 Patients

We retrospectively reviewed the clinical records of 282 patients with localized prostate cancer who underwent RP at our institution between July 2012 and July 2023. Informed consent was obtained from all patients. This study was approved by the ethical review board of our institution (36–005[12104]).

The use of VDS was initiated in May 2017; therefore, patients who underwent RP before this date did not receive prophylactic treatment. The IH occurrence data were updated, with the cutoff date for data inclusion being September 30, 2024. Patients with a history of bilateral IH repair or clinical IH detected during preoperative clinical examination were excluded.

IH was defined as the first point of unilateral or bilateral IH repair after RP. Previously reported risk factors, such as age, BMI, initial PSA, P‐vol, PLND, nerve sparing, pathological T‐stage, history of diabetes, pulmonary disease, hernia, operative methods and VDS, were considered potential risk factors in the statistical analysis.

### 2.2 Procedure

**FIGURE 1:**
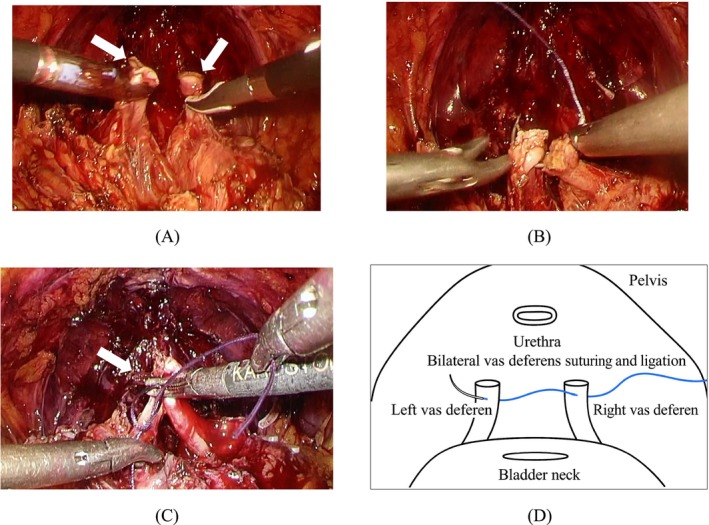
Representative images of the inguinal hernia prevention procedure during radical prostatectomy. (A) Vas deferens stumps after prostate removal (arrows). (B) Bilateral vas deferens sutured with a 3–0 absorbable suture. (C) Post‐suturing of both vas deferens (arrow). (D) Schematic of the vas deferens suture technique.

The patients underwent transperitoneal or open RRP. The type of procedure was chosen at the discretion of the attending physician. However, VDS was performed by a single surgeon. In the VDS group, after prostatectomy (Figure 1A), the bilateral vas deferens stumps were sutured using 3–0 absorbable thread (Figure 1B and D) and subsequently ligated (Figure 1C). In the control group, the stumps of the vas deferens remained free on both sides. Subsequently, anterior and posterior reconstructions were performed.^11^ Thereafter, urethra–vesical anastomosis and extended or limited PLND.^12^

### 2.3 Follow‐up

Patients were generally followed up every 3–4 months for the first 2 years after RP. Follow‐up included PSA measurements, physical examinations and questionnaires regarding whether patients had undergone IH surgery at another hospital. In addition, computed tomography scans were performed every six months. Thereafter, similar examinations were continued every six months, and from 5 years post‐surgery, these examinations were performed annually. If patients had symptoms of IH or if imaging revealed IH, we consulted the surgical department, and IH repair was performed when clinically indicated.

### 2.4 Statistical analysis

Continuous variables are reported as medians and interquartile ranges (IQRs), whereas categorical variables are reported as numbers and percentages. The patient characteristics presented in Table 1 were compared using an independent *t*‐test for numerical data (age, follow‐up period, BMI, PSA and P‐vol), and a chi‐square test or Fisher's exact test was performed for categorical data (PLND, nerve sparing, pathological T‐stage, Gleason score, diabetes, pulmonary disease, previous hernia surgery and prostatectomy approach used). The IH‐free rate was obtained using the Kaplan–Meier method and compared using the log‐rank test for each prognostic variable. Univariate and multivariate Cox regression analyses were performed to identify independent factors affecting the IH‐free rate.

**TABLE 1:** Baseline patient characteristics.

	Total	VDS (+)	Control	*P*‐value
**No of patients, n**	282	141	141	
**Age (year), median (IQR)**	71 (68–74)	72 (68–75)	71 (67–73)	0.021
**Follow‐up period (month), median (IQR)**	49 (1–143)	36 (1–84)	65 (3–143)	<0.001
**Body mass index (kg/m** ^**2**^ **), median (IQR)**	24.0 (22.3–25.8)	24.2 (22.4–25.5)	24.4 (22.2–26)	0.896
**Initial PSA level (ng/mL), median (IQR)**	7.0 (5.3–10.4)	6.9 (5.2–11.0)	7.0 (5.5–9.9)	0.342
**Prostate volume (cm** ^**3**^ **), median (IQR)**	29.4 (22.5–39)	29.5 (22.4–40)	29.4 (23–38.3)	0.312
**Lymph node dissection (%)**				<0.001
Yes	224 (79.4)	97 (68.8)	127 (90.1)	
No	58 (20.6)	44 (31.2)	14 (9.9)	
**Nerve sparing (%)**				0.059
Yes	40 (14.2)	26 (18.4)	14 (9.9)	
No	242 (85.8)	115 (81.6)	127 (90.1)	
**Pathological T‐stage (%)**				0.371
pT1–2	192 (68.1)	92 (65.2)	100 (70.9)	
pT3	90 (31.9)	49 (34.8)	41 (29.1)	
**Gleason score (%)**				0.314
≤ 7	220 (78.0)	106 (75.2)	114 (80.9)	
≥ 8	62 (22.0)	35 (24.8)	27 (19.1)	
**Diabetes, n (%)**				0.193
Yes	45 (16.0)	27 (19.1)	18 (12.8)	
No	237 (84.0)	114 (80.9)	123 (87.2)	
**Pulmonary disease, n (%)**				0.317
Yes	17 (6.0)	11 (7.8)	6 (4.3)	
No	265 (94.0)	130 (92.2)	135 (95.7)	
**Previous hernia operation, n (%)**				0.466
Yes	18 (6.4)	11 (7.8)	7 (5.0)	
No	264 (93.6)	130 (92.2)	134 (95.0)	
**Type of prostatectomy, n (%)**				<0.001
Open	151 (53.5)	54 (38.3)	97 (68.8)	
Laparoscopic	131 (46.5)	87 (61.7)	44 (31.2)	

Abbreviations: VDS; vas deferens suture, IQR; interquartile range.

All data were analysed using BellCurve statistical software (BellCurve Inc., Tokyo, Japan). Differences were considered significant if the two‐sided *P*‐value was < 0.05.

## 3 RESULTS

### 3.1 Patient characteristics

The 282 patients identified were assigned to either the VDS group (141 patients, operated between May 2017 and July 2023) or the control group (141 patients, operated between July 2012 and April 2017) (Table 1). The median patient age was 71 years (IQR, 68–74 years). With the data cutoff date set at September 30, 2024, the median follow‐up period of the VDS group (36 months, IQR: 23–55 months) was significantly shorter than that of the control group (65 months, IQR: 37–93 months).

The baseline demographic and clinical characteristics of the patients were similar between groups, except for the proportion of patients who underwent PLND and the type of prostatectomy. The follow‐up duration, PLND rate and RP approach differed significantly between groups (*P* < 0.001).

### 3.2 Clinical outcomes of VDS for prevention of IH after RP

**FIGURE 2:**
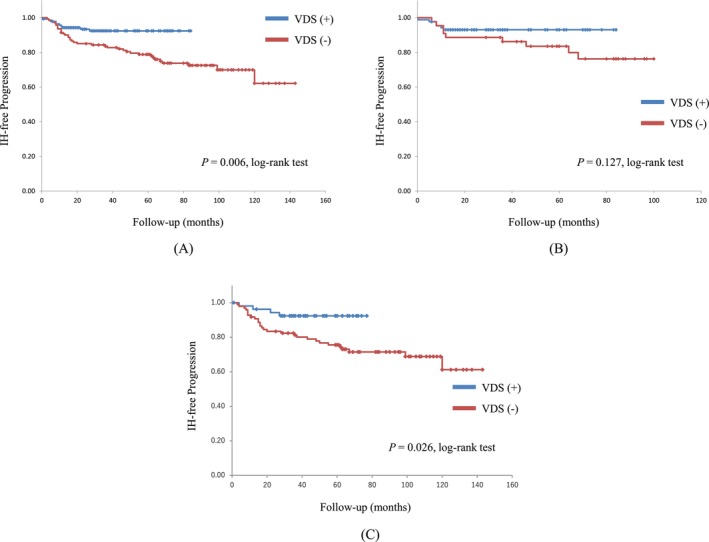
The Kaplan–Meier IH‐free rates after radical prostatectomy are shown in patients with or without VDS. (A) Total cohort. (B) Only laparoscopic radical prostatectomy. (C) Only retropubic radical prostatectomy. Abbreviations: VDS, vas deferens suture; IH, inguinal hernia.

Overall, IH developed in 10 (7.1%) and 37 (26.2%) patients in the VDS and control groups, respectively. The proportion of IH occurrence differed significantly between patients who underwent RP with VDS and those who underwent RP without VDS (*P* = 0.006) (Figure 2A). The median IH development duration was 15 months. The 2‐year IH‐free rates were 93.4% and 85.1% in the patients treated with and without VDS, respectively. The IH occurrence rate in patients who underwent RRP without VDS prophylaxis for postoperative IH was 28.8%, which was higher than the 20.5% observed in the patients who underwent LRP.

Among patients who underwent LRP, IH occurred in six (6.9%) and nine (20.5%) patients in the VDS and control groups, respectively. This represents a 33.7% relative risk reduction of IH after LRP in the VDS group compared with that in the control group. Although the VDS group tended to have a better IH‐free rate, no significant difference was observed (*P* = 0.127) (Figure 2B).

Among the patients who underwent RRP, IH occurred in four (7.4%) and 28 (28.8%) patients in the VDS and control groups, respectively. The difference in the IH‐free rate among patients who underwent RRP was also significant between the VDS and control groups (*P* = 0.026) (Figure 2C).

**FIGURE 3:**
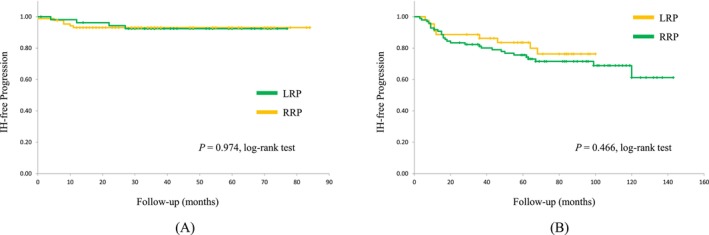
The Kaplan–Meier IH‐free rates of LRP versus RRP cases. (A) Patients who had undergone vas deferens suture as prophylaxis for IH after radical prostatectomy. (B) Patients who had undergone radical prostatectomy without any prophylaxis for IH. Abbreviations: LRP, laparoscopic radical prostatectomy; RRP, retropubic radical prostatectomy; IH, inguinal hernia.

In both the LRP and RRP cases, no significant differences were observed in the IH‐free rates between the VDS (*P* = 0.973) and control (*P* = 0.466) groups (Figure 3A and B).

We encountered no complications (e.g., seroma, testicular pain or neuralgia) specifically attributable to VDS in these patients.

### 3.3 Risk factors of IH

Table 2 shows the results of univariate and multivariate analyses using Cox regression analysis to identify the risk factors for postoperative IH. Among the putative risk factors tested, only VDS prophylaxis was associated with an IH‐free status after RP (hazard ratio [HR], 0.364; 95% confidence interval [CI], 0.179–0.744; *P* = 0.006) in the univariate analysis (Table 2). Similarly, in the multivariate analysis, VDS prophylaxis was the only independent predictor of IH development (HR, 0.36; 95%; CI, 0.177–0.734; *P* = 0.005) (Table 2).

**TABLE 2:** Univariate and multivariate analysis of factors for postoperative IH.

	Univariate		Multivariate	
Variables	HR (95%CI)	*P*‐value	HR (95% CI)	*P*‐value
Age	1.002 (0.948–1.059)	0.95		
BMI	0.905 (0.815–1.006)	0.063	0.902 (0.811–1.002)	0.055
PSA	0.991 (0.956–1.028)	0.637		
P‐vol	0.989 (0.967–1.01)	0.299		
PLND	0.706 (0.347–1.437)	0.337		
Nerve sparing	0.939 (0.398–2.216)	0.886		
T‐stage	0.657 (0.337–1.282)	0.218		
Gleason score	0.719 (0.352–1.47)	0.367		
Diabetes	0.879 (0.371–2.08)	0.769		
Pulmonary disease	0.724 (0.176–2.99)	0.656		
Previous hernia operation	2.007 (0.792–5.092)	0.142		
Type of prostatectomy	1.536 (0.824–2.857)	0.176		
VDS	0.364 (0.179–0.744)	0.006	0.36 (0.177–0.734)	0.005

Abbreviations: BMI, body mass index; P‐vol, prostate volume; PLND, pelvic lymph node dissection; VDS, vas deferens suture; HR, hazard ratio; CI, confidence interval.

## 4 DISCUSSION

IH, a postoperative complication associated with RP, is not life‐threatening, but it decreases the quality of life of patients as it creates a bulge in the groin area and causes pain or discomfort at the hernia site. Emergency surgeries are sometimes needed in cases of IH with severe pain or in cases of obstructive symptoms caused by incarceration or strangulation of the hernia sac contents.^13^ In addition, IH repair after RP is often difficult because of adhesions around the iliac vessels and internal inguinal ring.^14^, ^15^ Therefore, simple, safe and effective measures for preventing IH associated with RP are needed.

Alder et al. reviewed 54 studies involving 101 687 patients who underwent RRP, LRP or RALP. The estimated incidence of postoperative IH was 13.7%, 7.5% and 7.9%, respectively.^16^ They divided the reported IH prevention methods into four types. Two IH prevention methods, spermatic cord isolation and processus vaginalis transection, were investigated in the 12 studies. They found an IH incidence of 6.1% with the spermatic cord isolation method and 1.1% with the processus vaginalis transection method.^16^ Furthermore, Stranne et al. prospectively investigated a simple figure‐of‐8 suture in the internal ring to prevent postoperative IH and reported an IH incidence of 3.5% after RRP.^17^ Another prophylaxis method involved plugging the internal inguinal floor with hemostatic agents, which perfectly prevented IH after RALP.^18^ Alder et al. concluded that most studies on intraoperative IH prevention techniques demonstrated a substantially lower IH incidence in the intervention groups.^16^

Shimbo et al. compared non‐prevention and two types of prevention methods for post‐RALP IH.^14^ One method involved sufficient incision of the peritoneum around the internal inguinal ring, separation of the spermatic vessels and dissection of the vas deferens.^19^ The other method involved a modification of the first method, with added vas deferens transection.^14^ The group with vas deferens cutting demonstrated a statistically significantly higher IH‐free rate than did the group without vas deferens and the non‐prevention group.^14^ They suggested that the caudal shift in the bladder position due to RP causes medial traction on the internal inguinal ring via traction on the vas deferens and peritoneum.^19^ Medial traction on the internal inguinal ring then leads to the development of IH.^20^ Similarly, Kanda et al. reported that transection of the processus vaginalis with vas deferens dissection from the spermatic cord was superior to a simple prophylactic procedure involving only peritoneum dissection and isolation from the surrounding structures.^21^ These results imply that releasing the vas deferens without cutting it affects the development of post‐RP IH and supports our hypothesis that processus vaginalis migration into the internal inguinal ring by amputations of the vas deferens during RP causes post‐RP IH.

Kaiho et al. proposed that the mechanism underlying post‐RP IH involved the formation of a patent processus vaginalis when RP released tension placed on the vas deferens by the prostate.^22^ Therefore, separating the vas deferens from the peritoneum and/or transecting the processus vaginalis could prevent IH after RP.^22^ Our IH prevention method was the reverse of this proposal and represents a previously unreported concept. We hypothesized that cutting and releasing the vas deferens, which was originally fixed to the prostate during RP, would weaken the structure of the internal inguinal ring and surrounding tissue, resulting in IH. Therefore, by suturing both vas deferens, we aimed to maintain the integrity of the processus vaginalis and internal inguinal ring. Our hypothesis is compatible with previous proposals, and our procedure has been shown to be safe and effective in preventing IH‐associated RP.

We found no statistically significant differences between LRP and RRP cases with or without VDS, and also found no significant difference in the IH‐free rate in cases treated with LRP with or without VDS (Figure 2A, 3A and B). Our prophylactic procedure was more effective in the patients treated with RRP. In the prospectively controlled LAPPRO trial that investigated the risk of IH after RRP versus RALP, the relative risk of IH after RALP was not significantly lower (18%) than that after RRP. Furthermore, in a recent nationwide study, neither diagnosis nor hernia operation showed a substantial difference between RRP and RALP.^23^ Conversely, minimally invasive RP was associated with a significantly lower incidence of IH than RRP (HR, 0.82; 95% CI: 0.72–0.95; *P* = 0.007).^8^

Causative and risk factors associated with the development of IH after RP have been reported previously. They include advancing age, low BMI, small subcutaneous fat area, low psoas muscle volume, subclinical IH, previous hernia operation, low surgeon experience, patent processus vaginalis, non‐Retzius sparing RALP, nerve sparing, PLND, preoperative high international prostate symptom score and postoperative urinary straining.^9^, ^10^, ^22^, ^24^, ^25^, ^26^, ^27^, ^28^, ^29^, ^30^ These factors can be categorized into anatomical variations, patients' predispositions to IH, operative methods and perioperative abdominal pressure. In a review, Fernando et al. found that operative time, P‐vol, PSA and PLND were not associated with a substantial increase in IH incidence, while the role of the remaining factors was controversial.^10^ In the present study, no previously reported factors associated with IH development after RP were verified as risk factors in our multivariate analysis. In this study, only VDS prophylaxis was a negative and independent predictor of IH.

The present study had some limitations. First, this was a retrospective study with a relatively small sample size. Therefore, the findings need to be confirmed in a prospective study with a larger number of patients. Second, multiple surgeons performed RP procedures; however, only one surgeon performed VDS in all patients, ensuring the quality of the IH prevention step. Finally, several studies reported longer follow‐up periods than those in the present study, although others had similar observation periods.^6^, ^14^, ^20^, ^21^, ^22^, ^24^, ^27^

## 5 CONCLUSIONS

IH is a common complication of RP that reduces patients' quality of life and imposes economic burdens on society owing to the loss of work time and costs of IH repair. Urologists should consider IH a potential complication when performing RP for localized prostate cancer. Our study shows that the prophylactic VDS procedure is simple, safe and decreases the risk of IH after RP, making it worthy of consideration for patients undergoing RP.

## AUTHOR CONTRIBUTIONS

Kenichi Hata contributed to the design and the implementation of the research, data collection and management, data analysis and manuscript writing/editing. All authors were involved in administrative, technical or material support. Yuma Goto, Masaki Hashimoto, Yusuke Takahashi, Yuki Takiguchi, Yuya Iwamoto, Shun Saito, Ayaka Kawaharada, Yuki Enei, Keigo Sakanaka, Kazuhiro Takahashi, Akira Hisakane, Taisuke Yamazaki, Keiji Yasue, Soichiro Aoki, Kanako Kasai and Gen Ishii contributed to implementation of the research and were involved in the acquisition, analysis or interpretation of data. All authors were involved in the critical revision of the manuscript for important intellectual content. Takafumi Yanagisawa, Shunsuke Tsuzuki, Toshihiro Yamamoto, Hiroshi Sasaki, Jun Miki, Tatsuya Shimomura, Hiroki Yamada, Akira Furuta, Kenta Miki and Takahiro Kimura contributed to the project development and supervision.

## CONFLICT OF INTEREST STATEMENT

Takahiro Kimura was a paid consultant/advisor of Astellas, Bayer, Janssen and Sanofi. The other authors declare no conflicts of interest associated with this manuscript.

## Data Availability

The authors confirm that data supporting the findings of this study are available.
